# Fatal *Klebsiella pneumoniae* meningitis and concomitant disseminated intravascular coagulation in a patient with diabetes mellitus

**DOI:** 10.4103/0974-2700.50751

**Published:** 2009

**Authors:** Min-Po Ho, Kuang-Chau Tsai, Chun-Hsing Liao

**Affiliations:** Department of Emergency Medicine, Far Eastern Memorial Hospital, Taipei, Taiwan; 1 Department of Internal Medicine, Far Eastern Memorial Hospital, Taipei, Taiwan

**Keywords:** Death, diabetes mellitus, disseminated intravascular coagulation, *Klebsiella pneumoniae*, meningitis

## Abstract

Bacterial meningitis remains a major cause of death and long-term neurologic sequelae worldwide. We present a case of fatal *Klebsiella pneumoniae* meningitis and concomitant disseminated intravascular coagulation (DIC) in a 72-year-old woman with diabetes mellitus (DM). Both blood and cerebrospinal fluid cultures grew *Klebsiella pneumoniae*. Due to advanced age, newly recognized DM, *K. pneumoniae* bacteremia, and DIC, the prognosis of our patient was poor. Eight hours after arrival to the emergency department, cardiopulmonary resuscitation was necessary in this patient, but she died despite an early diagnosis and appropriate antibiotic therapy.

## INTRODUCTION

Bacterial meningitis is a life-threatening illness that required early diagnosis and aggressive treatment with appropriate antibiotic therapy. Without in-time diagnosis and treatment, it can be fatal. Here, we report a case of fatal *Klebsiella pneumoniae* meningitis and concomitant disseminated intravascular coagulation (DIC) in a patient with diabetes mellitus (DM).

## CASE REPORT

A 72-year-old woman presented to the emergency department (ED) with headache and neck pain that lasted 10 days. The patient's medical history was unremarkable. Mental status was alert. Initial vital signs included an arterial blood pressure 120/70 mmHg, heart rate 80/min, and body temperature 37°C. Initial neurological examination revealed neck stiffness and a positive Babinski sign. Her pupils measured 2.5 mm and were equal and reactive. A 12-lead electrocardiography (ECG) and chest X-ray were unremarkable. Abdominal ultrasonography showed no active lesions. She denied any systemic diseases, but her blood glucose was 514 mg/dl.

The patient's initial white cell count was 16030/mm^3^ with a left shift and 4% band form. Hemoglobin level was 13.1 mg/dL, and platelet count was 56000/mm^3^. Other abnormal laboratory findings were as follows: azotemia, metabolic acidosis, elevated erythrocyte sedimentation rate (ESR), prolonged prothrombin time, elevated fibrin degradation product, and positive D-dimer. The patient's overall clinical and laboratory findings clearly suggested the diagnosis of meningitis and concomitant DIC. Fluid resuscitation and intravenous administration of empirical antibiotics (ceftriaxone 2 g every 12 h and vancomycin 1 g every 12 h) were started. Emergent brain computed tomography (CT) scan showed no abnormalities. Lumbar puncture was performed, and cerebrospinal fluid (CSF) analysis showed 135 white cells/mm^3^ (89% neutrophils), protein 259 mg/dL, lactate dehydrogenase 89 IU/L, lactate 25.2 mmol, and glucose 112 mg/dL versus blood glucose 514 mg/dL.

Four hours later, the patient became deteriorated. Intubation and ventilation were initiated. Unfortunately, she developed cyanosis and ECG showed asystole 8 h after arrival to the ED. The patient received CPR including cardiac massage at the rate of 100/min with continuous ventilation, intravenous administration of epinephrine 1 mg every 3 min, and atropine 1 mg every 3 min (up to 3 mg), but she died despite aggressive treatment. Both blood and CSF cultures yielded *K. pneumoniae* that was susceptible to cephalosporins, aminoglycosides, and fluoroquinolones (and resistant to ampicillin).

## DISCUSSION

Despite the advent of new antibiotics and the improvement in clinical care techniques, bacterial meningitis remains a critical illness with a high morbidity and mortality.[1] A high incidence of DM among the adult patients with community-acquired spontaneous bacterial meningitis (38.5%) has been reported in Taiwan. *K. pneumoniae* was the most frequent causative pathogen followed by the streptococcal species.[2] *K. pneumoniae* was still the most common pathogen of adult bacterial meningitis, although its incidence has declined from 31.7% to 25.5%.[3] This change in causative pathogen might be explained by the increased number of postneurosurgical patients.

Diabetic patients vulnerable to disseminated *K. pneumoniae* infection should undergo further study since a higher percentage of patients with septic metastatic lesions have been those with diabetes.[4] In our patient, headache and neck stiffness were the chief complaints, but the patient did not experience fever for 10 days. Our patient initially showed evidence of bacterial meningitis and concomitant DIC with newly recognized diabetes mellitus. The patient's clinical course was fulminant and complicated by DIC. Despite early appropriate empirical antibiotic therapy, the patient's consciousness deteriorated and culminated in death.

In a study of 23 patients, 86.3% had significant concomitant bacteremia. The researchers postulated whether the intimal vascular abnormality seen in DM patients would predispose them to a hematogenous spread of *K. pneumoniae*, causing liver abscess and septic metastatic lesions.[4] In our case, the possible entry of *K. pneumoniae* to the central nervous system (CNS) might be due to hematogenous spread of *K. pneumoniae* bacteremia. The majority of metastatic infections (76.5%) occurred within the first 3 days after presentation. Unfortunately, antibiotics therapy dose not become fully effective during this time period.[5] The mortality rate for *K. pneumoniae* meningitis is reported to be in the range of 33.3% to 48.5%.[2,5]

Other risk factors for an unfavorable outcome for *K. pneumoniae* meningitis include advanced age, presence of otitis or sinusitis, absence of rash, a low score on the Glasgow Coma Scale on admission, tachycardia, a positive blood culture, an elevated ESR, thrombocytopenia, and a low CSF white cell count.[1] In our case, risk factors included advanced age, absence of rash, a positive blood culture, an elevated ESR, thrombocytopenia and a low CSF white cell count. Fang *et al.*,[6] revealed that a poor correlation between symptoms duration before therapy and outcome may be partially explained by the presence of preceding infection of extrameningeal *K. pneumoniae*. Since it difficult to determine the exact time when *K. pneumoniae* has invaded the CNS, it is also possible that some patients have a more fulminant course and deteriorate more quickly than others,[6] such as in our case.

*K. pneumoniae* meningitis and concomitant DIC is potentially lethal despite early appropriate empirical antibiotic therapy. Our patient, who also suffered from diabetes mellitus, was, unfortunately, an example of this. Early recognition and prompt, appropriate empirical antibiotic therapy is strongly recommended, even before CT scan and lumber puncture are performed.
